# Multilocus Sequence Typing (MLST) Genotypes of *Candida glabrata* Bloodstream Isolates in Korea: Association With Antifungal Resistance, Mutations in Mismatch Repair Gene (Msh2), and Clinical Outcomes

**DOI:** 10.3389/fmicb.2018.01523

**Published:** 2018-07-13

**Authors:** Seung A. Byun, Eun Jeong Won, Mi-Na Kim, Wee Gyo Lee, Kyungwon Lee, Hye Soo Lee, Young Uh, Kelley R. Healey, David S. Perlin, Min Ji Choi, Soo Hyun Kim, Jong Hee Shin

**Affiliations:** ^1^Department of Laboratory Medicine, Chonnam National University Medical School, Gwangju, South Korea; ^2^Department of Laboratory Medicine, University of Ulsan College of Medicine and Asan Medical Center, Seoul, South Korea; ^3^Department of Laboratory Medicine, Ajou University School of Medicine, Suwon, South Korea; ^4^Department of Laboratory Medicine, Yonsei University College of Medicine, Seoul, South Korea; ^5^Department of Laboratory Medicine, Chonbuk National University Hospital, Jeonju, South Korea; ^6^Department of Laboratory Medicine, Yonsei University Wonju College of Medicine, Wonju, South Korea; ^7^Public Health Research Institute, New Jersey Medical School-Rutgers, The State University of New Jersey, Newark, NY, United States

**Keywords:** *Candida glabrata*, MLST, *MSH2*, antifungal resistance, candidemia, mortality

## Abstract

*Candida glabrata* bloodstream infection (BSI) isolates from a particular geographic area have been reported to comprise a relatively small number of the major sequence types (STs) by multilocus sequence typing (MLST) analysis. Yet little is known about the characteristics of major ST strains of *C. glabrata*. To address this question in Korea, we investigated antifungal resistance and non-synonymous mutations of the mismatch repair gene (*msh2* mutations) in *C. glabrata* BSI isolates, as well as associated clinical characteristics, and compared the results according to MLST genotype. We assessed a total of 209 *C. glabrata* BSI isolates from seven hospitals in Korea for 2 years (2009 and 2014). Clinical features of candidemia and their outcomes were analyzed for 185 available cases. According to MLST, ST7 (47.8%) was the most common type, followed by ST3 (22.5%); the remainder represented 28 types of minor STs (29.7%). Fluconazole-resistance (FR) rates for ST7, ST3, and other strains were 9.0% (9/100), 8.5% (4/47), and 4.8% (3/62), respectively, and all were susceptible to amphotericin B and micafungin. All ST7 isolates harbored the V239L mutation in *msh2*, known to confer hypermutability, while 91.5% of ST3 isolates did not harbor the *msh2* mutation. Overall, isolates of the same ST had identical *msh2* mutations, with the exception of nine isolates. The *msh2* mutations were identified in 68.8% (11/16) of the FR isolates and 67.4% (130/193) of the fluconazole susceptible-dose dependent isolates. There was no significant difference in all clinical characteristics between ST3 and ST7. However, the 30-day mortality of *C. glabrata* candidemia due to the two major ST (ST3 or ST7) strains was significantly higher than that of candidemia due to other minor ST strains (45.1 vs. 25.0%, *p* < 0.05). Multivariate logistic regression analysis also showed that two major STs (ST3 and ST7) were independent predictors of 30-day mortality. This study showed for the first time that two STs (ST7 and ST3) were predominant among BSI isolates in Korea, and that *C. glabrata* BSI isolates belonging to two major MLST genotypes are characterized by higher mortality. In addition, most *msh2* mutations align with MLST genotype, irrespective of FR.

## Introduction

*Candida glabrata* is a commensal yeast in the human gastrointestinal tract, genitourinary tract, or oral cavity, but can cause serious bloodstream infections (BSI) in hospitalized patients. Compared with other common *Candida* species, *C. glabrata* has intrinsically low susceptibility to azole drugs, especially to fluconazole, but it can develop antifungal resistance rapidly in response to exposure to azoles or echinocandins (Pfaller et al., 2003; Barberino et al., 2006; Jensen et al., 2015). The frequency of BSI due to *C. glabrata* has increased significantly over the past two decades, and *C. glabrata* has become the second most commonly isolated bloodstream fungal pathogen in Europe and North America, with 10–30% of isolates being fluconazole resistant (FR) (Pfaller et al., 2007). However, geographic variation has been observed in both the frequency of isolation and fluconazole resistance among *C. glabrata* (Pfaller et al., 2010). *C. glabrata* is the fourth most common cause of candidemia, accounting for 14.2% of all cases of candidemia in Korea, and the incidence of *C. glabrata* candidemia and fluconazole resistance varies among hospitals (Won et al., 2015). Although the use of fluconazole is associated with an increased risk of BSIs caused by *C. glabrata* (Chow et al., 2008; Jensen et al., 2015; Won et al., 2015), many questions regarding the epidemiology of *C. glabrata* BSI remain unanswered (Lott et al., 2010).

*Candida glabrata* is a haploid organism in which a single DNA repair mutation is sufficient to cause an associated mutator phenotype and subsequent emergence of resistance-conferring mutations (Healey et al., 2016b). A recent study suggested that elevated levels of both triazole and multi-drug resistance associated with *C. glabrata* are at least partially due to the presence of loss-of-function mutations in the mismatch repair gene (*msh2* mutations) (Healey et al., 2016b). Other factors may also play a role, especially in the setting of low echinocandin and polyene resistance, as reported in France, where fluconazole resistance was not associated with the *MSH2* genotype pointing to other potential underlying factors (Dellière et al., 2016). Yet, genotype and *MSH2* status can be an important factor, as a recent multilocus sequence typing (MLST) study of clinical *C. glabrata* strains showed that different sequence types (STs) may carry different *msh2* alleles, suggesting that some determinants of drug resistance or virulence may also vary among STs (Healey et al., 2016a). MLST studies of *C. glabrata* BSI isolates conducted in the United States or China have indicated that *C. glabrata* BSI isolates comprise a relatively small number of the major STs, although these major STs may vary geographically (Lott et al., 2010, 2012; Hou et al., 2017). It is possible that major ST strains of *C. glabrata* possess higher virulence traits to cause BSIs than do other isolates, or different clinical characteristics. Until now, few studies have evaluated the microbiological and clinical characteristics of major ST strains of *C. glabrata* from a multicenter BSI surveillance study. The objectives of this study were to detect the major MLST genotypes among *C. glabrata* BSI isolates in Korean hospitals, and to evaluate whether *C. glabrata* BSI strains belonging to the major MLST genotypes have different clinical characteristics, and they display different rates of antifungal resistance or *msh2* mutations.

## Materials and Methods

### Microorganisms

A total of 209 non-duplicate isolates of *C. glabrata* recovered from blood cultures were included in this study. These isolates were recovered from seven Korean university hospitals during nationwide surveillance studies. Of the 209 BSI isolates, 94 were collected from September 2009 to August 2010 (period 1), and 115 were collected from January to December 2014 (period 2). All isolates were collected from blood cultures by routine methods in use in each laboratory. All *C. glabrata* isolates were submitted to Chonnam National University Hospital for testing. Species identification was based on colony morphology on CHROMagar *Candida* (BBL, Becton Dickinson, Sparks, MD, United States) at 35°C, and a commercial identification system (API 20C; bioMérieux, Marcy L’Etoile, France, or the Vitek 2 system; Vitek 2 ID-YST, bioMérieux). The isolates which did not belong to common STs in this study were re-identified by sequencing of the internal transcribed spacer (ITS) of ribosomal DNA and matrix-assisted laser desorption/ionization-time of flight mass spectrometry (MALDI–TOF Biotyper, Bruker Daltonics, Billerica, MA, United States) to differentiate from cryptic species within the *C. glabrata* complex (Lee et al., 2017). All isolates were confirmed as *C. glabrata*.

### Antifungal Susceptibility Testing

*In vitro* testing of susceptibility to fluconazole, voriconazole, amphotericin B, and micafungin was performed on all isolates using the Clinical and Laboratory Standards Institute (CLSI) broth microdilution (BMD) method, according to document M27-A3; minimum inhibitory concentrations (MICs) were determined after 24 h of incubation (Clinical Laboratory Standards Institute [CLSI], 2008). Species-specific clinical breakpoints proposed by the CLSI document M27-S4 were used for the categorical interpretation for fluconazole and micafungin MICs (Clinical and Laboratory Standards Institute [CLSI], 2012). In addition, the epidemiologic cutoff values (ECVs) were used for the threshold for resistance to voriconazole and amphotericin B, as the CLSI has not set clinical breakpoints for these two antifungal agents against *C. glabrata* (Pfaller and Diekema, 2012). Two reference strains, *Candida parapsilosis* ATCC 22019 and *Candida krusei* ATCC 6258, were included in each antifungal susceptibility test as quality control isolates.

### MLST

MLST was performed using a procedure described previously (Dodgson et al., 2003). The six genes selected for MLST analysis were *FKS*, *LEU2*, *NMT1*, *TRP1*, *UGP1*, and *URA3.* The reaction products were analyzed using an ABI Prism 3130xl Genetic Analyzer (Applied Biosystems). The allele profiles of the strains were defined according to the six MLST loci. Each unique allele profile was designated as a ST, determined using the MLST database.^1^

### Sequence Analysis of the *msh2* Gene

*Candida glabrata* genomic DNA was extracted as previously described (Dodgson et al., 2003), using the DNeasy Mini kit (Qiagen Inc., Valencia, CA, United States), and used as the template for amplification of the full-length *msh2* gene. Primers used for amplification and sequencing of *msh2* were as follows (Healey et al., 2016b): for amplification, 5′-CGATGAGCCGATCACTTTAC-3′ (CgMSH2u273F) and 5′-ACTTATCGAGGCACTCAGTC-3′ (CgMSH2d145R); for sequencing, 5′-ACCTAGCATCTCTTGTTCAC-3′ (CgMSH2c846R), 5′-CCTGGTCAATCTCATACACC-3′ (CgMSH2c1626R), 5′-CCATGCAAGTCCAAAACCATC-3′ (CgMSH2c2340R), and 5′-ACTTATCGAGGCACTCAGTC-3′ (CgMSH2d145R). Amplification conditions were as follows: an initial denaturation step for 3 min at 95°C, followed by 29 cycles of denaturation for 40 s at 95°C, annealing for 90 s at 56°C, elongation for 3 min at 72°C, and a final elongation step for 10 min at 72°C. Polymerase chain reaction (PCR) products were purified using a PCR purification kit (GeneAll Biotechnology, Seoul, Korea) according to the manufacturer’s recommendations. PCR products were sequenced using the abovementioned PCR primer pairs. Sequencing of both strands was performed using the ABI PRISM 3730XL Analyzer (Applied Biosystems, Foster City, CA, United States). Nucleotide sequences were analyzed using the MegAlign software package (DNAStar Inc., Madison, WI, United States) and compared with that of the database strain, CBS138/ XM_447585.

### Clinical Data Analysis

Clinical information including demographics, clinical status at positive culture, and therapy-related factors, and outcome of fungemia were collected retrospectively from 185 available patients with *C. glabrata* BSI (Kim et al., 2009; Choi et al., 2016). Clinical status at positive culture examined within 30 days before the candidemia occurrence included prior surgery, total parenteral nutrition use, prior antifungal exposure, immunosuppressive therapy, and neutropenia (defined as an absolute neutrophil count of 500/mm^3^). Severe sepsis, which was recently termed “sepsis” after the Sepsis-3 definition, was defined as life-threatening organ dysfunction caused by a dysregulated host response to infection and organ dysfunction identified as an acute change in total Sequential Organ Failure Assessment (SOFA) score (≥ 2 points) following infection (Singer et al., 2016). The “*Candida* score” was determined by variables including total parenteral nutrition, surgery, multifocal *Candida* colonization, and severe sepsis (Leon et al., 2006). The age-adjusted Charlson comorbidity index (ACCI) was calculated using the algorithm proposed by Charlson et al., in which 19 comorbid conditions are weighted and scored, with additional points added for age (Charlson et al., 1987; Hall et al., 2004). Patient outcome (survival or death) was assessed as all-cause mortality at 7, 30, 60, 90, and 120 days after the first positive blood culture result. This study was approved by the Institutional Review Board of Chonnam National University Hospital (IRB CNUH-2014-290). A waiver of the requirement for informed consent was granted given the retrospective nature of the project. Patient information was anonymized and de-identified prior to the analysis, and no information that could lead to patient identification was used.

### Statistical Analysis

Quantitative variables are expressed as means and standard deviation, and categorical variables as counts and percentages. Comparison of categorical variables between groups was performed using the χ^2^ or Fisher’s exact *t*-test. The independent *t*-test was used to compare continuous variables between two groups, the Mann–Whitney *U* test to compare continuous variables with non-normal distributions, and one-way analysis of variance (ANOVA) to compare variables among more than three groups, as appropriate (Jung et al., 2016). The predictive factors for 30-day mortality were analyzed using univariate and multivariate logistic regression analysis (Agresti, 2013). Variables with *p* < 0.1 in univariate analyses were included in multivariate regression models. The Cox proportional hazard model was used to evaluate potential risk factors for 30-day mortality by the hazard ratio (HR). The Kaplan–Meier method was used to calculate 120-day survival probability in subgroup analyses. All statistical analyses were performed using SPSS software (ver. 18.0; SPSS Inc., Chicago, IL, United States), and significance was determined at a level of *p* < 0.05.

## Results

### MLST and Antifungal Susceptibility

MLST results and the prevalence of FR among 209 BSI isolates of *C. glabrata* from 209 patients of seven Korean hospitals (hospitals A–G) spanning two time periods: September 2009 to August 2010 (period 1; *n* = 94), and January to December 2014 (period 2; *n* = 115) are shown in **Table 1**. Overall, 30 unique STs among the 209 clinical isolates were obtained. A total of 191 isolates belonged to the 12 previously described STs (2, 3, 6, 7, 10, 12, 19, 22, 26, 46, 55, and 59), whereas 18 isolates belonged to 18 new STs. Twenty STs were each derived from a single isolate, and ten STs were shared by 189 isolates (90.4%). ST7 (100 isolates, 47.8%) was the most common type, followed by ST3 (47 isolates, 22.5%), ST22 (12 isolates, 5.7%), and ST10 (9 isolates, 4.3%). ST7 represented 50.0% (47/94) and 46.1% (53/115) of all *C. glabrata* BSI isolates obtained during study periods 1 and 2, respectively, and ST3 represented 17.0% (16/94) and 27.0% (31/115) of all *C. glabrata* BSI isolates obtained during study periods 1 and 2, respectively. The major two ST types, ST7 and ST3, were found in all 7 hospitals through the study periods, however, the other STs were scattered in some hospitals. Overall, two major STs (ST7 and ST3) comprised 70.3% (147/209) of all *C. glabrata* BSI isolates obtained during the study periods. Of the 209 isolates, 16 (7.7%) were resistant to both fluconazole (MIC ≥ 64 μg/ml) and voriconazole (MIC > 0.5 μg/ml). All isolates were susceptible to amphotericin B (≤ 2 μg/ml) and micafungin (≤ 0.06 μg/ml). The FR rates were not significantly different among ST7, ST3, and other minor ST isolates, at 9.0% (9/100), 8.5% (4/47), and 4.8% (3/62), respectively. The FR rates were 5.3% (5/94) and 9.6% (11/115) during periods 1 and 2, respectively.

**Table 1:** Multilocus sequence typing (MLST) and fluconazole resistance (FR) status of 209 *Candida glabrata* bloodstream isolates from seven Korean hospitals.

Sequence type (ST) by MLST	Total (%), all periods	FR (%), all periods	Period^†^	No. of isolates (No. of FR isolates) in hospital							Subtotal (%), each period	Subtotal FR (%), each period
						
				A	B	C	D	E	F	G		
7	100 (47.8)	9 (9.0)	1	16	12 (2)	2	3	3	3 (1)	8	47 (50.0)	3 (6.4)
			2	23 (1)	9 (3)	4	6 (2)	3	4	4	53 (46.1)	6 (11.3)
3	47 (22.5)	4 (8.5)	1	6	1	1	3	1	3	1	16 (17.0)	
			2	9 (2)	9 (2)	5	2			6	31 (27.0)	4 (12.9)
22	12 (5.7)		1	2				2			4 (4.3)	
			2		2	1		2		3	8 (7.0)	
10	9 (4.3)		1	1	2	1		1			5 (5.3)	
			2	1	3						4 (3.5)	
55	5 (2.4)	1 (20.0)	1	1		1	1				3 (3.2)	
			2	2 (1)							2 (1.7)	1 (50.0)
2	4 (1.9)	2 (50.0)	1		1				2 (2)		3 (3.2)	2 (66.7)
			2	1							1 (0.9)	
6	4 (1.9)		1	1		1					2 (2.1)	
			2						2		2 (1.7)	
26	4 (1.9)		1			2					2 (2.1)	
			2					1		1	2 (1.7)	
12	2 (1.0)		1							1	1 (1.1)	
			2	1							1 (0.9)	
59	2 (1.0)		1	2							2 (2.1)	
			2								0 (0.0)	
Others^‡^	20 (9.6)		1	3	3			1		2	9 (9.6)	
			2	5	2			2	1	1	11 (9.6)	
Total	209 (100)	16 (7.7)	1	32	19	8	7	8	9	12	94 (100)	5 (5.3)
			2	42	25	10	8	8	7	15	115 (100)	11 (9.6)


*^†^Period 1: September 2009–August 2010; period 2: January 2014–December 2014. ^‡^Others include 20 STs, each derived from a single isolate.*

### Mutations in *msh2*

Non-synonymous mutations in the mismatch repair gene (*msh2* mutations) according to MLST genotype and fluconazole and voriconazole susceptibilities are described in **Table 2**. All 16 FR isolates and one FS (MIC, 32 μg/ml) isolate were resistant to voriconazole. *Msh2* mutations occurred in 65.1% (136/209) of the *C. glabrata* BSI isolates from Korean hospitals. *Msh2* mutations were detected in 68.8% (11/16) of FR isolates and 67.4% (130/193) of fluconazole susceptible-dose dependent (FS) isolates. Among the 189 BSI isolates that shared 10 STs (excluding 20 STs unique to a single isolate), isolates of the same ST harbored identical *msh2* mutations, with the exception of seven isolates: ST7 (three isolates), and ST3 (four isolates) showed an additional mutation compared with their ST strains. All 100 isolates of ST7 (nine FR and 91 FS) harbored the V239L mutation, associated with prominent hypermutability, and three isolates showed additional *msh2* mutations (V63F, V615A, or G204A). Among the 47 ST3 isolates, only four FS isolates harbored E7K (two isolates), Y167N (one isolate), or S118N (one isolate) mutations, and no non-synonymous mutations were found in 91.5% (43/47) of the ST3 isolates. All 12 ST22 isolates possessed the E456D mutation and all 9 ST10 isolates possessed the P208S/N890I mutation. No mutations were observed in any of the isolates of ST55 (five isolates), ST6 (four isolates), ST26 (four isolates), or ST59 (two isolates). Two isolates of ST12 harbored the E456D mutation. Overall, the *MSH2* sequence analysis revealed that the majority of isolates harbored the V239L (112 isolates, 53.6%), followed by no mutation (69 isolates, 33.0%), E456D (15 isolates, 7.2%), and P208S/N890I (9 isolates, 4.3%), respectively. *Msh2* mutations were found in 100% (100/100), 8.5% (4/47), and 59.7% (37/62) of ST7, ST3, and other isolates, respectively.

**Table 2:** Non-synonymous mutations in the mismatch repair gene (*msh2* mutations) in 209 *C. glabrata* bloodstream isolates according to MLST genotye, fluconazole and voriconazole susceptibilities.

MLST		*Msh2 mutation^∗^*	No. of isolates^§^				Total
			
ST	Allele profile^†^		Fluconazole susceptible-dose dependent	Fluconazole resistant	Voriconazole susceptible	Voriconazole resistant	
7	3-4-4-3-3-4	V239L	88	9	87	10	100
		V63F, V239L	1		1		
		V239L, V615A	1		1		
		G204A, V239L	1		1		
3	5-7-8-7-3-6	None	39	4	39	4	47
		E7K	2		2		
		Y167N	1		1		
		S118N	1		1		
22	7-5-6-3-1-8	E456D	12		12		12
10	8-4-3-5-1-2	P208S, N890I	9		9		9
55	3-6-22-2-3-9	None	4	1	4	1	5
2	1-2-2-1-1-1	V239L, A942T	2	2	2	2	4
6	2-5-7-5-1-2	None	4		4		4
26	7-4-3-4-1-8	None	4		4		4
12	7-5-6-12-1-8	E456D	2		2		2
59	7-13-17-9-3-19	None	2		2		2
Others^‡^		V239L	9		9		9
		E456D	1		1		1
		None	10		10		10
Total			193	16	192	17	209


*^†^*Allele profile for FKS-LEU2-NMT1-TRP1-UGP1-URA3.*^‡^Others include 20 STs which were each derived from a single isolate. *^∗^*The sequences of six non-synonymous mutations within MSH2 gene (E7K, V63F, S118N, Y167N, G204A, and V615A) found only in Korean isolates have been deposited in GenBank with accession numbers MH351625 to MH351627, and MH423282 to MH423284. ^§^ Fluconazole results were obtained by the CLSI clinical breakpoints (Clinical and Laboratory Standards Institute [CLSI], 2012), while the ECV of 0.5 μg/ml was used for the threshold for voriconazole resistance, as the CLSI has not set clinical breakpoints for voriconazole against C. glabrata (Pfaller and Diekema, 2012).*

### Clinical Characteristics and Mortality

A total of 185 patients were assessed to obtain data to predict clinical outcomes. Among all 185 patients, overall cumulative mortality occurred in 19.5, 39.5, and 47.0% at 7, 30, and 60 days after positive blood culture. **Table 3** summarizes the clinical characteristics of 185 patients infected with ST3, ST7, or the other minor ST BSI strains of *C. glabrata*. There were no significant differences among these three groups with respect to age, sex, or clinical status at positive culture. However, significant differences were found among the three groups in 30-day mortality rate (43.3, 48.8, and 25.0% for ST7, ST3, and minor STs, respectively; *p* = 0.035), and 60-day mortality rate (53.3% vs. 53.5% vs. 30.8%, *p* = 0.022, respectively), respectively. There were no significant differences among clinical characteristics between ST3 and ST7. When two major ST strains (ST7 and ST3) and other minor strains were considered, there were significant difference between two groups in neutropenia (two major STs vs. the minor STs; 9.0% vs. 0.0%, *p* = 0.025), 30-day mortality rate (two major STs vs. the minor STs; 45.1% vs. 25.0%, *p* = 0.012), 60-day mortality rate (two major STs vs. the minor STs; 53.4% vs. 30.8%, *p* = 0.006) and 120-day mortality rate (two major STs vs. the minor STs; 56.4% vs. 36.5%, *p* = 0.015; **Table 3**).

**Table 3:** Clinical characteristics of *C. glabrata* bloodstream isolates belonging to two major STs (ST7 and ST3) and other minor STs.

Variables	Two major MLST types			Other minor STs
		
	ST7	ST3	Total of two major STs	
No. of patients	90	43	133	52
**Demographics**				
Age, years, mean ± SD	68.2 ± 14.91	66.2 ± 14.22	67.5 ± 14.67	67.2 ± 12.23
Male, No. (%)	47 (52.2)	20 (46.5)	67 (50.4)	27 (51.9)
**Clinical status at positive culture**				
Prior fungal therapy, No. (%)	12 (13.3)	8 (18.6)	20 (15.0)	3 (5.8)
Total parenteral nutrition, No. (%)	41 (45.6)	20 (46.5)	61 (45.9)	21 (40.4)
Prior surgery (1 months), No. (%)	24 (26.7)	9 (20.9)	33 (24.8)	13 (25.0)
Severe sepsis, No. (%)	46 (51.1)	20 (46.5)	66 (49.6)	27 (51.9)
Neutropenia (< 500 cell/mm^3^), No. (%)	7 (7.8)	5 (11.6)	12 (9.0)^†^	0 (0.0)
Immunosuppressive therapy, No. (%)	22 (24.4)	8 (18.6)	30 (22.6)	8 (15.4)
ICU admission, No. (%)	19 (21.1)	12 (27.9)	31 (23.3)	9 (17.3)
Urine catheter, No. (%)	54 (60.0)	26 (60.5)	80 (60.2)	30 (57.7)
CVC use, No. (%)	52 (57.8)	31 (72.1)	83 (62.4)	26 (50.0)
Candida score, mean ± SD	1.5 ± 0.91	1.3 ± 0.86	1.4 ± 0.90	1.5 ± 1.35
ACCI, mean ± SD	6.6 ± 3.10	7.3 ± 2.89	6.9 ± 3.04	6.0 ± 2.81
**Therapy after positive culture**				
Antifungal therapy	64 (71.1)	28 (65.1)	92 (69.2)	33 (63.5)
Removal of CVC, No. (%)	40 (44.4)	26 (60.5)	66 (49.6)	24 (46.2)
**Clinical outcome**				
7-day all-cause mortality, No. (%)	18 (20.0)	11 (25.6)	29 (21.8)	7 (13.5)
30-day all-cause mortality, No. (%)	39 (43.3)	21 (48.8)	60 (45.1)^†^	13 (25.0)
60-day all-cause mortality, No. (%)	48 (53.3)	23 (53.5)	71 (53.4)^†^	16 (30.8)
90-day all-cause mortality, No. (%)	50 (55.6)	24 (55.8)	74 (55.6)^†^	19 (36.5)
120-day all-cause mortality, No. (%)	51 (56.7)	24 (55.8)	75 (56.4)^†^	19 (36.5)


*^†^*p*-value < 0.05, significant difference between two major STs (ST7 and ST3) vs. other minor STs. CVC, central venous catheter; ACCI, age-adjusted Charlson comorbidity index.*

**FIGURE 1:**
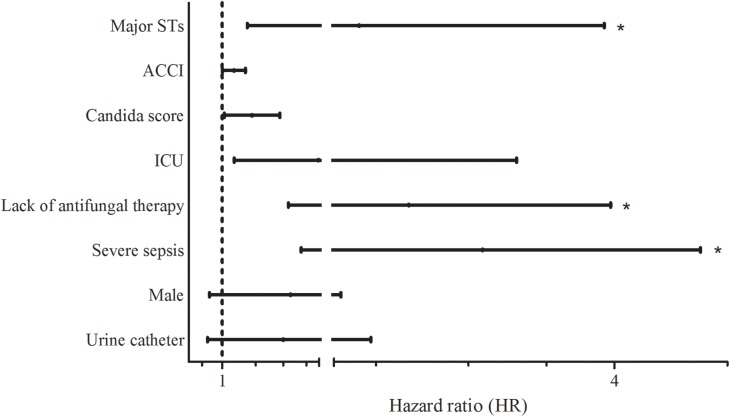
Hazard ratios (HRs) for 30-day mortality according to various factors. Lines indicate HRs and their 95% confidence intervals (CIs) for all variables suggested as prognostic factors for 30-day mortality by univariate analysis (*p* < 0.1) (**Table 4**). Asterisks indicate *p* < 0.05 in adjusted HRs. Abbreviations: Major STs, major sequence types (ST7 or ST3) by multilocus sequence typing (MLST; ACCI, age-adjusted Charlson comorbidity index; ICU, intensive care unit.

**FIGURE 2:**
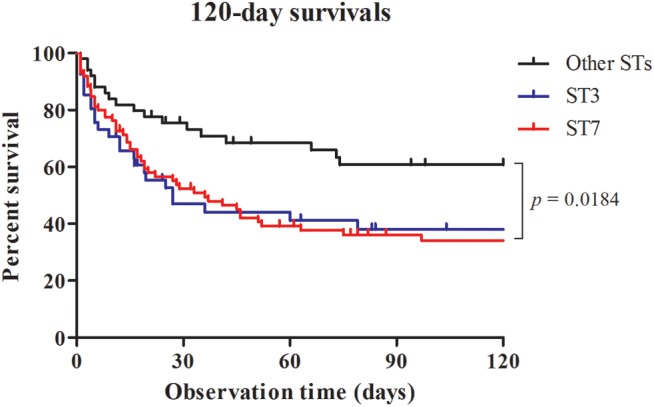
Survival analysis for candidemia patients. Patients with bloodstream infections were grouped according to MLST type (ST3, ST7, or Other STs) and censored at death or day 120. There was a significant difference in mortality rate between the curves.

**Table 4** shows the results of univariate and multivariate logistic regression analyses performed to identify risk factors for 30-day mortality. Based on the full multivariate model, the major STs (odd ratio [OR]: 4.058; 95% confidence interval [CI]: 1.349-12.201; *p* = 0.013), severe sepsis (OR: 9.886; 95% CI: 2.472-39.540; *p* = 0.001), and lack of antifungal therapy (OR: 2.850; 95% CI: 1.026–7.918; *p* = 0.044) were independent predictors of 30-day mortality. The adjusted Cox proportional hazard model revealed that stains with the two major STs (HR: 2.706; 95% CI: 1.236–5.921; *p* = 0.013) and severe sepsis (HR: 4.577; 95% CI: 1.884–11.119; *p* = 0.001) were significantly associated with 30-day mortality, in addition to lack of antifungal therapy (HR: 3.164; 95% CI: 1.733–5.777: *p* < 0.001), respectively (**Figure 1**). Kaplan–Meier survival analysis showed that the differences in mortality between patients with two major STs and those with other minor STs were sustained (*p* = 0.0067; log-rank tests). Patients with the two major STs experienced shorter survival than those with other minor STs (*p* = 0.0184 for 120-day survival; *p* = 0.0231 for 90-day survival; *p* = 0.0115 for 60-day survival; *p* = 0.0215 for 30-day survival; *p* = 0.0405 for 7-day survival; log-rank tests; **Figure 2**). There were no significant differences in clinical characteristics, including mortality rate, according to the *msh2* mutation type.

**Table 4:** Univariate and multivariate logistic regression analyses of prognostic factors for 30-day mortality.

Characteristic	Survivors (*N* = 112)	Non-survivors (*N* = 73)	Univariate analysis	Multivariate analysis^†^	
				
			*p*-value	Odd ratio (95% CI)	*p*-value
**Demographics**					
Age, years, mean ± SD	66.8 ± 14.99	68.4 ± 12.34			
Male, No. (%)	51 (45.5)	43 (58.9)	0.075	2.413 (0.934-6.229)	0.069
**Clinical status at positive culture**					
Prior antifungal exposure, No. (%)	11 (9.8)	12 (16.4)	0.195		
Intensive care unit admission, No. (%)^‡^	17 (15.2)	23 (31.5)	0.02	1.238 (0.403-3.800)	0.71
Total parenteral nutrition, No. (%)	46 (41.1)	36 (49.3)	0.253		
Immunosuppressive therapy, No. (%)	25 (22.3)	13 (17.8)	0.455		
Neutropenia, No. (%)	5 (4.5)	7 (9.6)	0.172		
Prior surgery, No. (%)	31 (27.7)	15 (20.5)	0.269		
Severe sepsis, No. (%)^‡^	41 (36.6)	52 (71.2)	< 0.001	9.886 (2.472-39.540)	0.001
CVC use, No. (%)	66 (58.9)	43 (58.9)	0.97		
Urine catheter, No. (%)^‡^	60 (53.6)	50 (68.5)	0.036	1.973 (0.573-6.792)	0.281
Candida score, mean ± SD^‡^	1.3 ± 0.85	1.7 ± 1.24	0.009	0.729 (0.359-1.480)	0.381
ACCI, mean ± SD^‡^	6.3 ± 2.84	7.2 ± 3.16	0.045	1.053 (0.889-1.246)	0.551
**Therapy after positive culture**					
Removal of CVC, No. (%)	52 (46.4)	38 (52.1)	0.246		
Lack of antifungal therapy, No. (%)^‡^	29 (25.9)	31 (42.5)	0.019	2.850 (1.026-7.918)	0.044
***C. glabrata* isolate**					
Major STs (ST 7 or ST 3)^‡^	73 (65.2)	60 (82.2)	0.01	4.058 (1.349-12.201)	0.013
Fluconazole resistance	6 (5.4)	7 (9.6)	0.277		


*^†^Variables with *p* < 0.1 by univariate analysis were evaluated using multivariate analysis. ^‡^Variables with *p* < 0.05 between survivors and non-survivors. CVC, central venous catheter; ACCI, age-adjusted Charlson comorbidity index.*

## Discussion

To our knowledge, this is the first study to report that two major ST (ST7 and ST3) strains, comprising 70.3% of all *C. glabrata* BSI isolates from Korean hospitals, are linked to higher mortality in candidemia patients. The rates of antifungal resistance were not significantly different among ST7, ST3, and other strains. Of the two major STs, ST7 isolates harbored the same V239L substitution in *msh2*, which is associated with hypermutability, whereas nearly all ST3 isolates lacked *msh2* mutations. Overall, we found that occurrences of most *msh2* mutations were associated with specific MLST genotypes, irrespective of FR. The present study highlights that MLST genotypes are associated not only with particular *msh2* mutations but also with the prediction of mortality in candidemia patients with *C. glabrata*.

To date, only a few studies have investigated the MLST genotypes of BSI isolates of *C. glabrata* collected by multicenter surveillance (Lott et al., 2010, 2012; Hou et al., 2017). MLST of 230 BSI isolates in three United States cities showed that three STs (ST3, ST16, and ST19) were the most abundant, together comprising 51% of all BSI isolates (Lott et al., 2010). The prevalence of ST16 among BSI isolates of *C. glabrata* in Atlanta-area hospitals increased significantly between 1992 and 1993 (4%) and 2008 (40%). An increase was also observed for ST3, from 19 to 28%, but the prevalence of ST19 did not change (Lott et al., 2010). A recent study from China showed that ST7 and ST3 were the most common STs (68.0 and 9.0% of all 200 BSI isolates, respectively; Hou et al., 2017). In the present study, ST7 and ST3 were the predominant genotypes (47.8 and 22.5% of all BSI isolates, respectively) among 209 BSI isolates. During the two study periods, the prevalence of ST7 remained unchanged at 4.5-year intervals (50.0 and 46.1%, respectively), but that of ST3 increased (17 and 27%, respectively), suggesting that ST3 strains evolved for optimal adaptation to new environments as causative agents of BSI in Korea. No ST16 strain was isolated in either of two studies in China (Hou et al., 2017) or Korea (present study). Collectively, our results support the notion that the major MLST genotypes of *C. glabrata* BSI isolates vary both geographically and over time.

Clinical characteristics of the specific MLST genotypes of *C. glabrata* BSI isolates has not previously been reported. In the present study, cumulative mortalities of *C. glabrata* BSI at 7 and 30 days after the first episode of candidemia were 19.5 and 39.5%, respectively, which is similar to previous reports (Lortholary et al., 2014; Ding et al., 2015). Notably, we found a significant difference in the 30-day mortality of *C. glabrata* candidemia due to the two major ST (ST3 or ST7) strains and in that of candidemia due to other minor ST strains (45.1% vs. 25.0%). There were no significant differences in all clinical characteristics between ST3 and ST7. We could not find significant differences in other clinical features between patients with either of the two major STs or other minor STs, except for neutropenia. Although neutropenia may have an impact on the mortality of *C. glabrata* fungemia (Wu et al., 2017), only 12 patients (6.5%) had neutropenia in the present study. We found the same results that two major STs were associated with higher 30-day mortality than other minor STs, even after we removed the data for the 12 patients with neutropenia.

Multivariate logistic regression identified the two major STs, severe sepsis, and lack of antifungal therapy as independent variables associated with a higher 30-day mortality rate. The adjusted Cox proportional hazard model also revealed that the two major STs and severe sepsis were significantly associated with 30-day mortality, in addition to lack of antifungal therapy. Proper administration of antifungal agents (Bassetti et al., 2017) or therapeutic measures such as antifungal treatment within the first 48 h (Garnacho-Montero et al., 2017), severe sepsis and septic shock (Puig-Asensio et al., 2014; Garnacho-Montero et al., 2017) have been reported as variables independently associated with 30-day mortality of candidemia. However, candidemia due to the two major ST strains of *C. glabrata* was more likely to be fatal than candidemia due to other strains in our cohort. The Kaplan–Meier survival analysis also showed that the differences in mortality between the major STs and the other minor STs were sustained from 30 to 120 days after positive blood culture. Although it is difficult to establish that the death of a patient was attributable solely to candidemia, a link between the presence of two ST strains and increased mortality seems the likely explanation for our data.

A recent study reported that *msh2* mutations varied according to MLST genotypes; however, only 48 clinical isolates were tested (Healey et al., 2016a). In our work, we compared *msh2* mutations by MLST genotype using 209 BSI isolates from seven hospitals during a 2-year period, and demonstrated that most isolates of the same ST harbored identical *msh2* mutations. Among the 189 BSI isolates that shared 10 STs (excluding 20 STs unique to a single isolate) in the present study, isolates of the same ST harbored identical *msh2* mutations, with the exception of nine isolates that showed an additional mutation compared to their ST strains. In a previous report, strains harboring V239L/A942T or V239L in *msh2* were identified in centers in the United States, Switzerland, Qatar, and India, but the majority of strains isolated from United States medical centers exhibited E231G/L269F or P208S/N890I mutations (Healey et al., 2016a). In the present study, the majority of isolates (53.6%) harbored V239L, followed by no mutation (33.0%), E456D (7.2%), and P208S/N890I (4.3%). Our data suggest that geographic differences in the frequency of *msh2* mutations in clinical *C. glabrata* isolates (Healey et al., 2016b) may reflect differences in the distribution of the major MLSTs of *C. glabrata* among hospitals or geographic locales.

It has been reported that *msh2* mutations are more frequent among FR (65%) and multidrug resistant (62%) *C. glabrata* isolates than among FS isolates (52%) (Healey et al., 2016b). However, among the 209 BSI isolates in the current study, the frequency of *msh2* mutations was similar between FS (67.4%, 130/193) and FR (68.8%, 11/16), which is consistent with the findings of a recent study (Dellière et al., 2016). In the present study, the rate of *msh2* mutations was different among ST7 (100%), ST3 (8.5%), and other (58.1%) strains, but FR rates did not differ significantly among ST7 (9.0%), ST3 (8.5%), and other (4.8%) strains. It is possible that not all *msh2* mutations are equal and they may not all contribute to the acquisition of fluconazole resistance. Notably, V239L and P208S/N890I, but not E456D or E7K, were shown to confer partial loss of *msh2* function (Dellière et al., 2016; Healey et al., 2016b); how the other *msh2* polymorphisms identified within our study influence phenotype has yet to be determined. However, a more recent study has shown the absence of azole or echinocandin resistance in *C. glabrata* isolates in India despite background prevalence of strains with defects in DNA mismatch repair pathway; albeit, isolates were collected from patients with limited antifungal exposure. Notably, 49% of tested strains in India contained partial loss of *msh2* mutation (Singh et al., 2018). Also, Healey et al. showed that additional mechanisms of DNA repair, including other MMR genes, and other cellular systems may influence the mutagenic potential of *C. glabrata* strains (Dellière et al., 2016). Taken together, these data suggest that *msh2* mutations in *C. glabrata* isolates occur independently of FR acquisition, and the *msh2* mutation alone is not responsible for the acquisition or increased prevalence of FR in specific STs.

Clinical outcomes of fungemia are dependent on the fungus (virulence and susceptibility), host status (severity of the underlying condition), and therapy (timing in relation to infection stage, antifungal drug choice and dosing; Arendrup, 2013). In the present study, we found that two MLST genotypes more frequently caused BSI infections than other genotypes in Korea, and mortality at 30 days was higher for BSI with two major ST strains than for BSI with other strains, suggesting that the pathogenic potential of *C. glabrata*, with respect to significant morbidity and mortality, may partly depend on the MLST genotype. The most likely explanation for our results is that the two major ST strains may be more virulent than other minor ST strains in Korean patients. Therefore, some of the virulence traits of *C. glabrata* which include growth at 37°C, high stress resistance, resistance to starvation, drug resistance, high adherence or presence of several auxotrophies (Gabaldon and Carrete, 2016) require investigation for strains of major MLST genotypes. In addition, further study of major MLST genotypes of *C. glabrata* BSI isolates and their expansion over time will contribute significantly to our understanding of incidence rates and patient outcomes of *C. glabrata* BSI, and their antifungal resistance in particular geographic locations.

## Author Contributions

SK, WL, M-NK, KL, HL, and YU participated in isolate and clinical data collection. SB, EW, and MC carried out the experimental studies and performed the statistical analysis. KH and DP helped draft the manuscript. SB, EW, and JS conceived the study, participated in study design and data analysis, and were responsible for writing and submission of the final manuscript. All authors read and approved the manuscript.

## Conflict of Interest Statement

The authors declare that the research was conducted in the absence of any commercial or financial relationships that could be construed as a potential conflict of interest.

## Footnotes

1 http://www.mlst.net/
